# Maximum Entropy and Theory Construction: A Reply to Favretti

**DOI:** 10.3390/e20040285

**Published:** 2018-04-14

**Authors:** John Harte

**Affiliations:** Energy and Resources Group, and Department of Environmental Science, Policy & Management, University of California at Berkeley, Berkeley, CA 94720, USA; jharte@berkeley.edu

**Keywords:** maximum entropy, ecology, macroecology, abundance, metabolism, ecological theory

## Abstract

In the maximum entropy theory of ecology (METE), the form of a function describing the distribution of abundances over species and metabolic rates over individuals in an ecosystem is inferred using the maximum entropy inference procedure. Favretti shows that an alternative maximum entropy model exists that assumes the same prior knowledge and makes predictions that differ from METE’s. He shows that both cannot be correct and asserts that his is the correct one because it can be derived from a classic microstate-counting calculation. I clarify here exactly what the core entities and definitions are for METE, and discuss the relevance of two critical issues raised by Favretti: the existence of a counting procedure for microstates and the choices of definition of the core elements of a theory. I emphasize that a theorist controls how the core entities of his or her theory are defined, and that nature is the final arbiter of the validity of a theory.

The maximum entropy theory of ecology (METE) predicts the shapes of probability distributions describing patterns in the abundance, metabolism, and spatial distribution of individuals and taxonomic groups in ecosystems [1,2,3,4,5]. It does so across a wide range of spatial scales, across a wide diversity of habitats (e.g., forests, alpine meadows, deserts) and types of taxa (e.g., birds, trees, forbs, mammals, arthropods, microorganisms; species, genera, families, …), and without adjustable parameters. In ecosystems that are relatively undisturbed by human activity or by natural processes such as fire and landslides, the predictions are remarkably accurate [1,2,3,6,7] but in ecosystems that are responding to major anthropogenic disturbance the predictions tend to fail [3,4,8].

Among the successfully-predicted metrics are the Fisher logseries distribution of abundances over species [1,3], the distribution of metabolic rates over individuals [1,3], the spatial abundance distribution of individuals within species [1,3], the species–area relationship [2,3], the relationship between the abundance of a species and the average metabolic rate of its species [1,3], and the structure of taxonomic trees (distribution of lower-level taxa within higher taxonomic groupings) [5].

Favretti [9] claims that a correct application of MaxEnt would, in contrast to METE, predict a geometric distribution both for abundances over the species and metabolic rates over individuals; moreover, again in contrast to METE, it would predict the absence of correlation between the abundances of species and the metabolic rates of the individuals within the species. 

I summarize here what the core entities and definitions are for METE, and discuss the relevance of two critical issues raised by Favretti: the existence of a counting procedure for microstates and the choices of definition of the core elements of a theory. I argue that Favretti’s model demonstrates that one can construct other MaxEnt-based ecological theories that result in predictions different from METE. However, I disagree with his conclusion that, because his model is based on an explicit state-counting procedure, METE must be incorrect. The issue over which I disagree with Favretti lies at the heart of the meaning of theory in science, and in particular with his implicit characterization of the process of theory construction.

MaxEnt is an inference procedure that selects the least informative (maximum information entropy) probability distribution that is compatible with prior knowledge, thereby minimizing bias [10]. The use of MaxEnt to construct scientific theory entails three stages [4]:

In stage one, the phenomena that one wishes to predict are identified. In the case of METE, those phenomena are the ones described by the collection of metrics listed in the second paragraph above. In METE, these predicted distributions are not directly calculated using MaxEnt but rather from certain core distributions that are chosen at Stage 2.

In Stage 2, one defines the core probability distributions, and their independent variables, whose forms will be predicted using MaxEnt. Those core distributions are selected in a manner that allows the shapes of the metrics in stage one to be derived. In the case of METE, those core distributions include what is termed “the ecological structure function”, *R*(*n*,*ε*|conditionals). It is defined (I thank Dr. Favretti for pointing out an error in the way the structure function is defined in some publications and for clarifying the correct definition.) by a two-step process [1]. In the first step, choose a species from the species pool. In second step, choose an individual from the pool of all individuals that are found in species with abundance *n*. The structure function is the joint probability that the chosen species has abundance *n* and the chosen individual has metabolic rate between *ε* and *ε* + d*ε*. METE includes other core distributions [3,5] but the structure function defined above is the one that is relevant to my disagreement with Favretti.

In Stage 3, constraints on the defined core distributions in Stage 2 are selected. These constraints comprise the prior knowledge, and appear as the “conditionals” in the core distributions. Information entropy is then maximized subject to these constraints using the method of Lagrange multipliers. In the case of METE, the constraints are the average abundance per species (*N*_0_/*S*_0_) and the average metabolic rate per species (*E*_0_/*S*_0_).

With Stages 1–3 completed, the MaxEnt inference procedure [10] predicts, using the method of Lagrange multipliers and the choice of Shannon information entropy (see below), the form of the structure function and the other core distributions that comprise METE. From those distributions, the predicted forms of the ecological metrics such as the species abundance distribution and the degree of correlation between abundance and metabolic rate follow with straightforward mathematics [3,4]. This procedure results in, among other predictions, a logseries abundance distribution and a negative correlation between abundance and metabolic rate. No error has been found in the actual calculation that maximizes the Shannon entropy of the structure function *R*(*n*,*ε*|conditionals), subject to the above constraints.

Favretti shows that an alternatively-constructed theory yields markedly different predictions, and in particular predicts no correlation between abundance and metabolic rate. He chooses as his core distribution a function *P*(*n*,*ε*) rather than the ecological structure function *R*(*n*,*ε*) used in the formulation of METE. From the way in which *P* is defined, the metabolic constraint on the function *P* is a specified value of the mean metabolic rate per individual, *E*_0_/*N*_0_, whereas the metabolic constraint on the ecological structure function defined in METE is mean metabolic rate per species, *E*_0_/*S*_0_. Indeed, it is from the way the METE structure function *R* is defined that the appropriate constraint on metabolic rates is the mean metabolic rate per species. Thus, at both Stages 2 and 3, METE differs from the Favretti model. It is not surprising that they yield different predictions. But which, if either, is correct?

Favretti asserts that his is correct because his predicted form for *P*(*n*,*ε*) can be derived from a specific microstate counting argument. This extrapolates to ecology the classical state-counting combinatoric argument that results in a Boltzmann distribution for molecular energies. In contrast, the METE calculation makes no explicit assumption about microstate combinatorics, but this raises very interesting questions. First, is the METE result, and, in particular, the existence of correlation, incompatible with state counting? Lest it be thought that the absence of correlation is an inevitable feature of any set of distributions derived by maximizing log(*W*), where *W* is the number of microstates, we note that a combinatorics-based ecological model [11] has been constructed that does indeed yield the correlation predicted by METE: an inverse relationship between metabolism and abundance. Importantly, that model examined combinatoric possibilities under a continuum of levels of distinguishability of the individuals within species (from distinguishable, as in classical particles, to non-distinguishable, as in quantum particles). In the limit of non-distinguishable individuals within species, the state counting procedure introduced in [11] results in exactly the correlation between abundance and metabolism that is predicted by METE.

The biological significance of this finding is under investigation, and while we do not claim literal indistinguishability for individual organisms within species, we suspect that the success of the microstate-counting argument in [11] may have something to do with the fact that within-species genetic differences are far smaller than between species differences. I emphasize that we do not currently know whether the microstate counting derivation in [11] will yield not only the correlations predicted by METE but, in addition, all its many other predictions.

It should be noted, however, that while the Boltzmann microstate counting argument does yield the same distribution as does Jaynes’s MaxEnt inference procedure in many situations, most famously the energy distribution of molecules in an ideal gas; the Jaynes procedure is more general and is not explicitly tied to any particular scheme for counting microstates, or to any assumptions about the identity of entities at the microscale, or the thermodynamic limit of a system. MaxEnt simply uses the method of Lagrange multipliers to find the smoothest, flattest form of a probability distribution, or, in other words, the form that maximize information entropy, and is compatible with prior knowledge (see, for example, [12] for an excellent review of the broad generality of the MaxEnt inference procedure).

A remaining question raised by [9] concerns the justification for the use of Shannon information entropy. It is well known that the Shannon information entropy function is the unique function that satisfies a certain set of axioms, but other entropy functions exist under different assumptions about the fundamental axioms. Lacking first-principles knowledge of which set of axioms apply in ecology, I have examined the outcomes if METE is formulated using alternative choices of the information entropy function (Tsallis and Renyi). Those measures are characterized by a parameter q, where the limit q goes to 1 and returns the Shannon entropy function. Empirically, values of q that deviate from 1 by more than a few percent result in predictions for macroecological metrics that are demonstrably false [3]. I emphasize that this is an empirical, not a fundamental, justification for the use of Shannon information entropy.

If you define an ecological theory the way it is defined in METE (the three stages specified above), then a consistent set of predictions for the ecological metrics can be rigorously derived from the structure function, and any observer using MaxEnt correctly, with Shannon information entropy, will arrive at the same conclusions. Those conclusions will generally differ if the theory is constructed differently, as in Favretti’s model, but that is not pertinent to the consistency of METE.

I speculate that the empirical success of the choices METE makes at Stages 1–3 above is linked to the important role of evolution in ecology, and to the fact that intraspecific differences among individuals are small compared to interspecific differences. In particular, the usefulness of the METE definition of the structure function and the choice of constraints may have to do with the concept of an equilibrium fitness landscape and the equalization of fitness across species, not across individuals. This notion is supported, additionally, by the success of a generalized version of METE [5] that extends the theory to higher taxonomic levels (with additional state variables corresponding to numbers of genera, families, etc.), and generalizes the definition of the structure function and the constraints accordingly.

To summarize, METE uses the method of Lagrange multipliers to maximize the Shannon information entropy of a well-defined distribution that is a function of well-defined independent variables, subject to well-defined constraints. METE is mathematically consistent and yields numerous predictions about the abundance, distribution, and energetics of species and individuals in undisturbed ecosystems. By redefining the core elements of METE, Favretti has constructed an ecological model that, not surprisingly, yields predictions differing from those of METE. Both models cannot simultaneously make correct and accurate predictions for the reason given by Favretti [9]. Most importantly, METE predicts correlation between abundance and metabolism, whereas Favretti does not. Each of these two differing models can be derived by maximizing the Shannon information entropy of a differently-defined core distribution, using the prior knowledge in a way that is consistent with the differences in the definitions of the core distributions (structure functions). Favretti argues that his model is correct, and METE is not, because the former is derivable from a microstate-counting argument. Whether all of METE’s many predictions, and not just its predicted correlation between abundance and metabolism, are derivable from a microstate counting argument remains to be seen, but the MaxEnt inference procedure, while compatible with a state-counting derivation in many instances, is more general. The empirical success of METE, and not of the Favretti model, suggests that there may be merit in the way METE is constructed, but the larger point is that the originator of a theory has control over how a theory is defined.
